# Maintenance Decision-Making Using Intelligent Prognostics Within a Single Spare Parts Support System

**DOI:** 10.3390/s25030837

**Published:** 2025-01-30

**Authors:** Bowei Zhang, Changhua Hu, Jianfei Zheng, Hong Pei

**Affiliations:** College of Missile Engineering, Rocket Force University of Engineering, Xi’an 710025, China

**Keywords:** maintenance decision-making, single spare parts support system, spare parts ordering, replacement

## Abstract

Health management is the foothold of remaining useful life (RUL) prediction, known as ‘prognostics’. However, sudden failures in complex systems can lead to increased downtime and maintenance costs, ultimately diminishing system health and availability. Considering intelligent prognostics of components, maintenance decision-making for spare parts ordering and replacement is proposed within a spare parts support system. The decision-making process aims to minimize costs while maximizing availability as its primary objective. It considers spare parts ordering time and replacement time as key decision variables. By developing a maintenance decision-making model, it aims to determine the optimal time for ordering and replacing spare parts. This maintenance approach is designed to provide technical support for effective and rational equipment management decision-making.

## 1. Introduction

In the era of the Industrial Revolution 4.0, new concepts have emerged alongside this technological shift, particularly predictive maintenance (PM). PM plays a crucial role in sustainable manufacturing and production systems by introducing a digital model for machine maintenance [1,2,3,4,5,6,7,8,9]. The advancement of sensing technology and artificial intelligence has led to an exponential increase in data extracted from production processes. PM effectively minimizes machine downtime and associated costs, maximizes machine life cycles, and enhances the overall quality and pace of production. This method typically involves a precise workflow that begins with understanding the project and collecting data and concludes with the decision-making stage [10,11,12,13,14,15,16,17,18,19].

## 2. Literature Review

The previous work of PM focuses on a large number of monitoring data generated during the operation of complex systems, and much information can be obtained through fault diagnosis and intelligent prognostics. Among them, the prediction information includes the probability density function (PDF), cumulative distribution function (CDF), and reliability function (RF) of RUL. Based on RUL prediction information, maintenance staff can carry out condition-based maintenance for complex systems, formulate reasonable health management activities in advance for problems that may occur during operation, and implement preventive and remedial measures in advance [20,21,22,23,24].

In the industrial sector, particularly in the field of aircraft key equipment (such as engines and gyroscopes), numerous researchers have conducted comprehensive studies. Zheng et al. [25] introduced an adaptive maintenance decision-making approach that utilizes RUL prediction information for nonlinear degraded systems. This method effectively determines the optimal replacement time and dynamic monitoring intervals while considering constraints such as operating costs and system availability. Building on this foundation, Mu et al. [26] integrated spare parts ordering time as a variable within the maintenance decision-making process, optimizing both spare parts replacement and ordering times. This approach facilitates more sophisticated maintenance activities compared to focusing on a single objective. Following this, Zheng et al. [27] expanded the maintenance decision-making model by incorporating the influence of condition monitoring for condition-based maintenance and spare parts ordering of individual component systems. They employed a normal distribution to characterize the effects of periodic condition assessments on equipment degradation. Using RUL prediction information, they established a maintenance decision-making model aimed at minimizing the long-term average costs associated with component maintenance.

Recent studies have also explored various methodologies for maintenance decision-making. For instance, Errandonea et al. [28] conducted a literature review on digital twins for maintenance, highlighting the potential of digital twins in enhancing predictive maintenance strategies. Similarly, Fink et al. [29] discussed the potential, challenges, and future directions for deep learning in prognostics and health management applications, emphasizing the importance of advanced machine learning techniques in improving maintenance decision-making. These studies demonstrate the potential of integrating advanced machine learning techniques into maintenance decision-making processes.

In the context of spare parts inventory management, research has shown that optimizing inventory strategies can significantly reduce downtime and maintenance costs. For example, Meddaoui et al. [30] established reliable methods for predicting failures in manufacturing excellence, demonstrating the benefits of predictive maintenance in reducing downtime and improving system reliability. Additionally, Molęda et al. [31] reviewed maintenance approaches for the power industry, highlighting the transition from corrective to predictive maintenance and its impact on system reliability.

The C-MAPSS dataset has been widely used in the field of predictive maintenance to validate the effectiveness of various models. Li et al. [32] proposed a new data-driven prediction method based on deep convolutional neural networks, which achieved high prediction accuracy for RUL estimation on the C-MAPSS dataset. This study highlights the potential of deep learning techniques in handling large-scale industrial data for predictive maintenance.

Despite these advancements, current research primarily focuses on scenarios without spare parts inventory support. It emphasizes multiple constraints, such as operating costs and availability, as objectives for making maintenance decisions and ordering spare parts [33,34]. However, real-world situations often involve sudden failures of complex system components, which can significantly increase the risk of downtime and maintenance costs, putting the system in an unhealthy state. To minimize the time required for component replacement, reduce downtime risks, and enhance system availability, there is a pressing need for an operational strategy for spare parts inventory management. This strategy aims to extend the operational lifespan of the system.

This article presents a maintenance decision-making method designed to operate within a single spare parts support system. The aim is to prevent prolonged system shutdowns caused by unexpected failures and to ensure system availability. The proposed method utilizes intelligent prognostics from the well-known C-MAPSS dataset. It establishes a multi-objective joint decision-making model that takes cost and availability into account simultaneously. The goal is to maximize the availability of components while minimizing maintenance costs. This approach helps determine the optimal time for spare parts ordering and component replacement.

The structure of this article is organized as follows: Section 1 provides a brief introduction, while Section 2 provides a more detailed literature review. Section 3 defines the single spare parts support system and discusses the associated challenges. Section 4 introduces the proposed maintenance decision-making model. In Section 5, a numerical verification analysis is conducted. Finally, Section 6 summarizes the key points of the article.

## 3. Problem Description and Definition

To ensure the high availability of the entire system, this article investigates a decision-making method for maintenance concerning the ordering and replacement of spare parts. It focuses on a single spare parts support system that relies on intelligent prognostics. The discussion includes an overview of the single spare parts support system within the context of a single inventory spare parts component operation system, as well as various maintenance decision-making scenarios related to replacement and spare parts ordering based on specific scenarios.

### 3.1. Single Spare Parts Support System

The single spare parts support system, often referred to as the single inventory spare parts single component operation system, is designed for a solitary large, valuable, and replaceable service component with the capability for single inventory management. To minimize system downtime and enhance overall operational efficiency, the inventory of spare parts should be maintained at a minimum of one.

**Figure 1:**
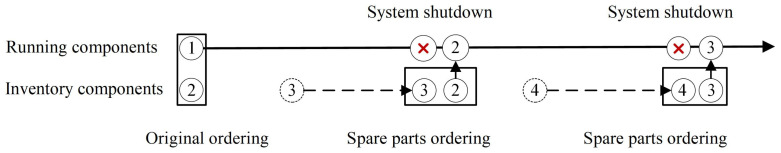
Single spare parts support system.

As illustrated in Figure 1, during the actual operating cycle, two components of the same model are initially purchased at the same time. One component is designated for active use, while the other serves as an inventory spare. The inventory component is stored in the warehouse and can be swiftly deployed to replace the operational component in the event of an unexpected failure, thereby restoring the system’s normal operation. At any given time, one component is actively in use, accompanied by an additional redundant part in storage. Furthermore, considering the pre-ordering of spare parts for inventory components is essential. This approach ensures that even if the original component fails and is replaced by an inventory spare, a backup spare part remains available in stock, preventing a shortage due to potential failures of newly replaced components.

Within the operational life cycle of a component, the minimum single spare parts inventory system primarily focuses on the cost associated with ordering spare parts. It considers ordering just one component in the later stages, with the exception of ordering two components simultaneously during the initial stage. To clarify, the operation of ordering two components for the first time is referred to as the ‘original ordering’, while the subsequent ordering of a single component is termed the ‘spare parts ordering’.

### 3.2. Intelligent Prognostics

Obtaining the RUL is essential for making informed maintenance decisions regarding degraded equipment. For effective intelligent prognostics, it is important to provide interval estimates of prediction results. In recent years, traditional deep learning models that incorporate dropouts have proven capable of capturing the uncertainty inherent in prediction results, allowing for the provision of interval estimates. By training the RUL prediction network using a training set and subsequently inputting new condition monitoring (CM) data, we can derive the corresponding RUL prediction results. For convenience, the PDF of the predicted RUL is simplified as $f\left(l_{k}\left|X_{1:k}\right.\right)$, where $l_{k}$ is the predicted RUL at time $t_{k}$ and $X_{1:k}$ is the input CM data. According to the PDF of predicted RUL, the cumulative distribution function (CDF) and reliability function (RF) of predicted RUL can be obtained as follows:

(1) $$\begin{array}{l} F\left(l_{k}\left|X_{1:k}\right.\right)=\int_{0}^{l_{k}}f\left(\tau \left|X_{1:k}\right.\right)d\tau \\ R\left(l_{k}\left|X_{1:k}\right.\right)=1-F\left(l_{k}\left|X_{1:k}\right.\right) \end{array}$$

### 3.3. Maintenance Description

**Figure 2:**
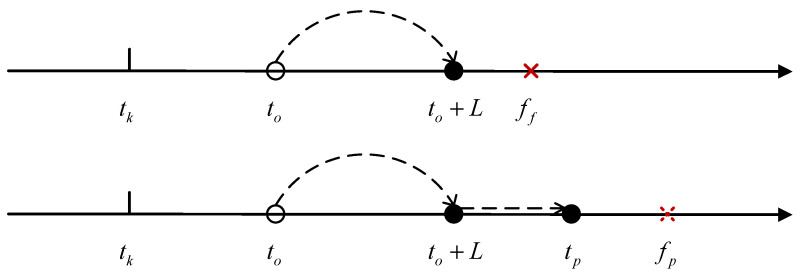
Two possible scenarios throughout the life cycle.

According to the information above, two potential scenarios may arise throughout the entire lifecycle of the components, as illustrated in Figure 2. Let $t_{k}$ be the current CM time and $t_{k+1}$ be the next CM time. $t_{o}$ and $t_{p}$ are the predicted spare part ordering time and replacement time, respectively. L is the delivery time for spare parts. Replacement is divided into failure replacement $f_{f}$ and preventive replacement $f_{p}$, and $f_{f}<f_{p}$.

Scenario 1: If the component has been ordered according to the predicted ordering time and has arrived, but it fails before the predicted replacement time, the existing spare parts must be replaced immediately. This means that if the component suddenly fails, there will be costs associated with maintaining the inventory of spare parts as well as costs for ordering new spare parts.

Scenario 2: If the parts have been ordered according to the predicted ordering time and have arrived, and the original parts have not reached their predicted replacement time, then the existing spare parts will be replaced according to the scheduled replacement time. In this case, if the parts suddenly fail, there will again be costs related to maintaining the inventory of spare parts and costs associated with ordering new ones.

## 4. Methodology

**Figure 3:**
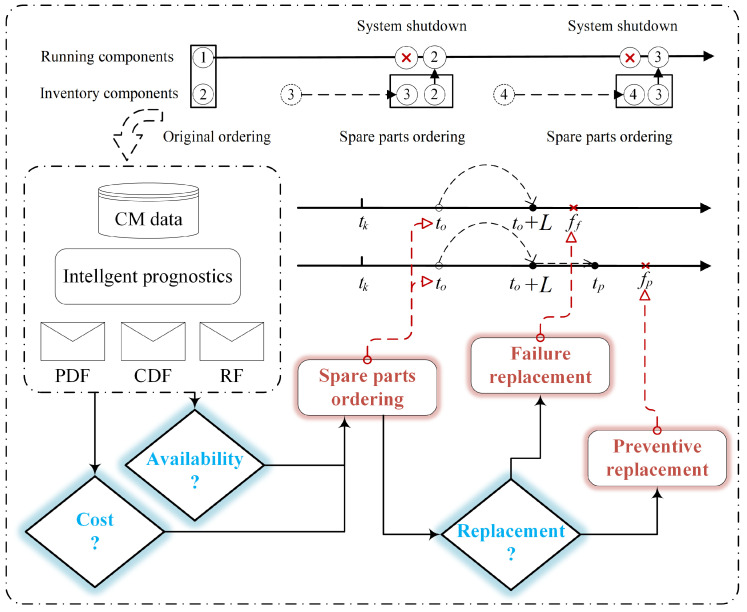
The flowchart of the maintenance decision-making method.

The flowchart illustrating the maintenance decision-making method is presented in Figure 3. Initially, within the single spare parts support system, intelligent prognostics utilizing condition monitoring (CM) data can extract information such as the probability density function (PDF), cumulative distribution function (CDF), and reliability function (RF). Subsequently, a long-term average cost model and a long-term average availability model are collaboratively developed to determine the optimal time for ordering spare parts. Finally, a replacement time point is identified, taking into account both failure replacement and preventive replacement scenarios.

It is necessary to make the following assumptions based on the two scenarios described in the previous section.

(1) The condition monitoring interval is fixed, and the cost of a single monitoring is $C_{m}$. The equipment requires preventive replacement with a cost $C_{p}$ and failure replacement with a cost $C_{f}$. We assume $C_{m}<C_{p}<C_{f}$.

(2) The state monitoring behavior of the original spare parts service will not affect parts of residual life, and redundancy of spare parts storage in warehouse degradation is negligible. The time that completes the failure replacement and preventive replacement is $T_{f}$ and $T_{p}$, respectively.

(3) A period of time L between ordering and arrival of a spare part is considered to be fixed.

(4) Let $C_{o}$ be the ordering cost of the spare part, $C_{h}$ be the cost of maintaining inventory per unit time, and $C_{c}$ be the the total cost of maintaining inventory parts.

(5) Let CH be the inventory cost, TH be the inventory time, and CR be the replacement cost, including the failure replacement cost and preventive replacement cost.

### 4.1. Long-Term Average Cost Model

Based on the model assumptions in the previous section and the renewal–reward theorem [35,36], the long-term average cost model can be expressed as

(2) $$C_{k}\left(t_{o},t_{p}\right)=\frac{EU}{EV}$$

where the EU represents the expected cycle cost, the EV represents the expected cycle length, and $t_{o}$ and $t_{p}$ are the spare parts ordering and parts replacement time to be optimized, respectively.

The first step is to derive the EU, which can be expressed as

(3) $$EU=C_{o}+C_{c}+C_{H}+C_{R}+kC_{m}$$

Total cost of spare parts ordering: since the quantity of spare parts to be ordered in a life cycle is 1, the total cost of spare parts ordering is $C_{o}$.

Total maintenance cost of inventory parts: there are long-term maintenance costs of inventory parts in the early stages of scenario 1 and scenario 2. The total inventory time is divided into two parts, which are calculated as follows:

(4) $$C_{c}=t_{k}\times C_{h}+(t_{k}-t_{o}-L)\times C_{h}=(2t_{k}-t_{o}-L)\times C_{h}$$

where the inventory maintenance time of spare parts is from the start of operation to the current monitoring time $t_{k}$. Similarly, the inventory maintenance time of ordered spare parts is $t_{k}-(t_{o}+L)$.

In order to get the total cost of inventory, you need to derive the corresponding inventory time first. Inventory time exists in scenario 1 and scenario 2.

(5) $$\begin{array}{cc} T_{H} & =\int_{t_{o}+L}^{t_{p}}\left[l_{k}-\left(t_{o}+L\right)\right]\cdot f\left(l_{k}\left|X_{1:k}\right.\right)dl_{k}+\int_{t_{p}}^{\infty }\left[t_{p}-\left(t_{o}+L\right)\right]\cdot f\left(l_{k}\left|X_{1:k}\right.\right)dl_{k}\\ & =\int_{t_{o}+L}^{t_{p}}\left[l_{k}-\left(t_{o}+L\right)\right]dF\left(l_{k}\left|X_{1:k}\right.\right)+\int_{t_{p}}^{\infty }\left[t_{p}-\left(t_{o}+L\right)\right]dF\left(l_{k}\left|X_{1:k}\right.\right)\\ & =\left[l_{k}-\left(t_{o}+L\right)\right]\cdot F\left(l_{k}\left|X_{1:k}\right.\right){|}_{t_{o}+L}^{t_{p}}\\ & -\int_{t_{o}+L}^{t_{p}}F\left(l_{k}\left|X_{1:k}\right.\right)dl_{k}+\left[t_{p}-\left(t_{o}+L\right)\right]\cdot \left[1-F\left(t_{p}\left|X_{1:k}\right.\right)\right]\\ & =\left[t_{p}-\left(t_{o}+L\right)\right]-\int_{t_{o}+L}^{t_{p}}F\left(l_{k}\left|X_{1:k}\right.\right)dl_{k}\\ & =\int_{t_{o}+L}^{t_{p}}\left[1-F\left(l_{k}\left|X_{1:k}\right.\right)\right]dl_{k}\\ & =\int_{t_{o}+L}^{t_{p}}R\left(l_{k}\left|X_{1:k}\right.\right)dl_{k} \end{array}$$

Therefore, the inventory cost is

(6) $$C_{H}=C_{h}\cdot T_{H}=C_{h}\int_{t_{o}+L}^{t_{p}}R\left(l_{k}\left|X_{1:k}\right.\right)dl_{k}$$

The replacement cost includes preventive replacement cost and ineffective replacement cost, which can be expressed as

(7) $$C_{R}=C_{p}\left[1-\int_{0}^{t_{p}}f\left(l_{k}\left|X_{1:k}\right.\right)dl_{k}\right]+C_{f}\int_{0}^{t_{p}}f\left(l_{k}\left|X_{1:k}\right.\right)dl_{k}$$

Overall, the expected cycle cost is

(8) $$\begin{array}{cc} EU & =C_{o}+C_{c}+C_{H}+C_{R}+kC_{m}\\ & =C_{o}+t_{k}\times C_{h}+(t_{k}-t_{o}-L)\times C_{h}+C_{h}\int_{t_{o}+L}^{t_{p}}R\left(l_{k}\left|X_{1:k}\right.\right)dl_{k}\\ & +C_{p}\left[1-\int_{0}^{t_{p}}f\left(l_{k}\left|X_{1:k}\right.\right)dl_{k}\right]+C_{f}\int_{0}^{t_{p}}f\left(l_{k}\left|X_{1:k}\right.\right)dl_{k}+kC_{m}\\ & =C_{o}+(2t_{k}-t_{o}-L)\times C_{h}+C_{h}\int_{t_{o}+L}^{t_{p}}R\left(l_{k}\left|X_{1:k}\right.\right)dl_{k}\\ & +C_{p}\left[1-\int_{0}^{t_{p}}f\left(l_{k}\left|X_{1:k}\right.\right)dl_{k}\right]+C_{f}\int_{0}^{t_{p}}f\left(l_{k}\left|X_{1:k}\right.\right)dl_{k}+kC_{m} \end{array}$$

Step two, derive the EV. In the two possible scenarios that may occur throughout the entire life cycle of the component, scenario 1 involves the spare parts arriving but the component failing before the predicted preventive replacement time; therefore, immediate failure replacement is carried out. Scenario 2 involved the spare parts arriving and the components failing within the predicted replacement time, in which case, preventive replacement should be carried out at the predicted replacement time. Based on the above analysis, the EV can be expressed as

(9) $$\begin{array}{cc} EV & =t_{k}+\int_{t_{o}+L}^{t_{p}}\left(l_{k}+T_{f}\right)\cdot f\left(l_{k}\left|X_{1:k}\right.\right)dl_{k}+\int_{t_{p}}^{\infty }\left(t_{p}+T_{p}\right)\cdot f\left(l_{k}\left|X_{1:k}\right.\right)dl_{k}\\ & =t_{k}+\left(t_{p}+T_{f}\right)\cdot F\left(t_{p}\left|X_{1:k}\right.\right)-\\ & \int_{t_{o}+L}^{t_{p}}F\left(l_{k}\left|X_{1:k}\right.\right)dl_{k}+\left(t_{p}+T_{p}\right)-\left(t_{p}+T_{p}\right)F\left(t_{p}\left|X_{1:k}\right.\right)\\ & =t_{k}+\left(T_{f}-T_{p}\right)\cdot F\left(t_{p}\left|X_{1:k}\right.\right)-\int_{t_{o}+L}^{t_{p}}F\left(l_{k}\left|X_{1:k}\right.\right)dl_{k} \end{array}$$

By combining Formulas (8) and (9), the long-term average cost model can be obtained as follows:

(10) $$\begin{array}{cc} C_{k}\left(t_{o},t_{p}\right) & =\frac{EU}{EV}\\ & =\frac{C_{o}+(2t_{k}-t_{o}-L)\times C_{h}+C_{h}\int_{t_{o}+L}^{t_{p}}R\left(l_{k}\left|X_{1:k}\right.\right)dl_{k}}{t_{k}+\left(T_{f}-T_{p}\right)\cdot F\left(t_{p}\left|X_{1:k}\right.\right)-\int_{t_{o}+L}^{t_{p}}F\left(l_{k}\left|X_{1:k}\right.\right)dl_{k}}\\ & +\frac{C_{p}\left[1-\int_{0}^{t_{p}}f\left(l_{k}\left|X_{1:k}\right.\right)dl_{k}\right]+C_{f}\int_{0}^{t_{p}}f\left(l_{k}\left|X_{1:k}\right.\right)dl_{k}+kC_{m}}{t_{k}+\left(T_{f}-T_{p}\right)\cdot F\left(t_{p}\left|X_{1:k}\right.\right)-\int_{t_{o}+L}^{t_{p}}F\left(l_{k}\left|X_{1:k}\right.\right)dl_{k}} \end{array}$$

### 4.2. Long-Term Average Availability Model

Availability is an important indicator for measuring the actual operational efficiency of a component, used to describe the probability or expected time occupancy of the component being able to operate normally within a certain period of time. The construction form is as follows:

(11) $$A_{k}\left(t_{o},t_{p}\right)=\frac{EO}{EV}$$

where the EO represents the expected running time of the component, and the EV represents the expected cycle length, which is the sum of the running time and downtime of the component. Among the four possible scenarios in the whole life cycle of components, scenario 1 and scenario 2 have downtime caused by out-of-stock and replacement activities because spare parts have not arrived at the time of component failure. Scenario 1 has downtime due to failure replacement, and scenario 2 has downtime due to preventive replacement.

The first step is to derive the expected running time, which can be expressed as

(12) $$\begin{array}{cc} EO & =t_{k}+\int_{t_{o}+L}^{t_{p}}l_{k}\cdot f\left(l_{k}\left|X_{1:k}\right.\right)dl_{k}+\int_{t_{p}}^{\infty }t_{p}\cdot f\left(l_{k}\left|X_{1:k}\right.\right)dl_{k}\\ & =t_{k}+t_{p}\cdot F\left(t_{p}\left|X_{1:k}\right.\right)-\left(t_{o}+L\right)\cdot F\left(t_{o}+L\left|X_{1:k}\right.\right)\\ & -\int_{t_{o}+L}^{t_{p}}F\left(l_{k}\left|X_{1:k}\right.\right)dl_{k}+t_{p}-t_{p}\cdot F\left(t_{p}\left|X_{1:k}\right.\right)\\ & =t_{k}+t_{p}-\left(t_{o}+L\right)\cdot F\left(t_{o}+L\left|X_{1:k}\right.\right)-\int_{t_{o}+L}^{t_{p}}F\left(l_{k}\left|X_{1:k}\right.\right)dl_{k} \end{array}$$

By combining Formula (9) and Formula (12), the long-term average availability can be obtained:

(13) $$\begin{array}{cc} A_{k}\left(t_{o},t_{p}\right) & =\frac{EO}{EV}\\ & =\frac{t_{k}+t_{p}-\left(t_{o}+L\right)\cdot F\left(t_{o}+L\left|X_{1:k}\right.\right)-\int_{t_{o}+L}^{t_{p}}F\left(l_{k}\left|X_{1:k}\right.\right)dl_{k}}{t_{k}+\left(T_{f}-T_{p}\right)\cdot F\left(t_{p}\left|X_{1:k}\right.\right)-\int_{t_{o}+L}^{t_{p}}F\left(l_{k}\left|X_{1:k}\right.\right)dl_{k}} \end{array}$$

### 4.3. Multi-Objective Maintenance Decision-Making Model

In order to solve the multi-objective optimization problem, a decision boundary is constructed. Specifically, let the long-term average availability meet the requirements of component use efficiency in engineering practice and let the long-term average cost be minimized on this basis. Then, the multi-objective maintenance decision-making model can be rewritten as

(14) $$\begin{array}{l} Minimize\;\;\;\;\;\;\;\;C_{k}\left(t_{o},t_{p}\right)\\ Subject\;to\;\;\;\;\;\;\;A_{k}\left(t_{o},t_{p}\right)\geq \zeta \\ \;\;\;\;\;\;\;\;\;\;\;\;\;\;\;\;t_{k}\leq t_{o}\leq t_{p} \end{array}$$

where ζ is the availability threshold, which is usually set according to the actual operation requirements of the project. In order to ensure that the parts meet the availability requirements, the spare parts ordering and parts replacement time are within a certain value range. The optimal spare parts ordering and component replacement time can be calculated using Equation (15).

(15) $$\begin{array}{c} {t_{o}}^{*},{t_{p}}^{*}=\arg\;\min\;C_{k}\left(t_{o},t_{p}\right)\\ \;t_{o},t_{p}\in \left[t_{\min},t_{\max}\right] \end{array}$$

## 5. Numerical Verification

### 5.1. Data Description on C-MAPSS Dataset and Intelligent Prognostics

This section selects a well-known C-MAPSS dataset released by NASA to verify the method. The dataset consists of multiple multivariate time series, including three operating settings and 21 types of sensors that have a significant impact on engine performance. The specific information is shown in Table 1. This dataset has four sub-datasets, and the sub-dataset named FD001 is used for our case study. Each sub-dataset is further divided into training engines and testing engines. In the training engines, the entire time series of each engine from normal operation until system failure is recorded. Conversely, in the test engines, the time series for each engine ends sometime before the system fails.

**Figure 4:**
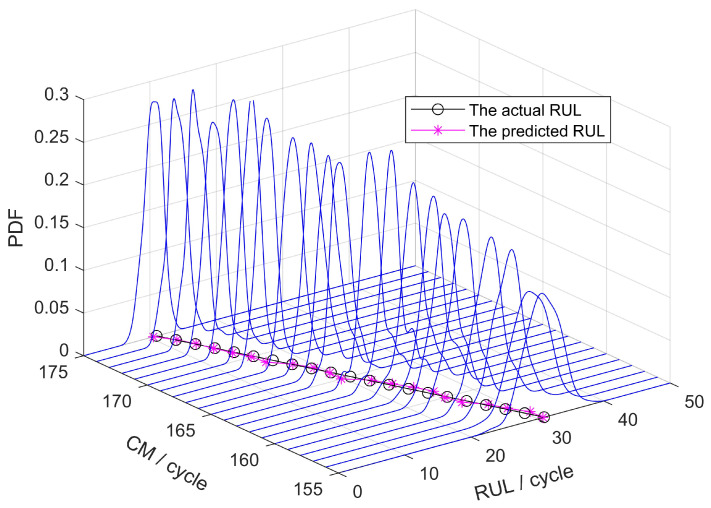
PDF for different CM cycles of RUL cycles.

This article presents the results of a new deep learning prediction method based on the Temporal Convolutional Network (TCN) and Bidirectional Long Short-term Memory (BiLSTM) neural networks, as referenced in [37]. Among them, TCN mainly undertakes feature extraction and captures short-term local dependencies, while BiLSTM mainly undertakes long-term memory and captures long-term macro dependencies. Figure 4 illustrates the PDF for the 76th engine among the tested engines. The RUL prediction method demonstrates strong predictive performance and offers practical reference value.

### 5.2. Results on the Testing Data of FD001 at the 76th Engine

Based on the above intelligent prognostics, the parameters in the multi-objective maintenance decision-making model are set as $C_{p}=10000\$$, $C_{f}=30000\$$, $C_{m}=30\$$, $C_{o}=1000\$$, $C_{i}=10\$$, $T_{f}=15\;h$, $T_{p}=9\;h$ and availability threshold ζ= 0.98. The No. 76 engine has CM data for 176 CM cycles. It is assumed that the interval between each CM cycle is 3 h, and the lead time noted L of spare parts is 72 h.

**Figure 5:**
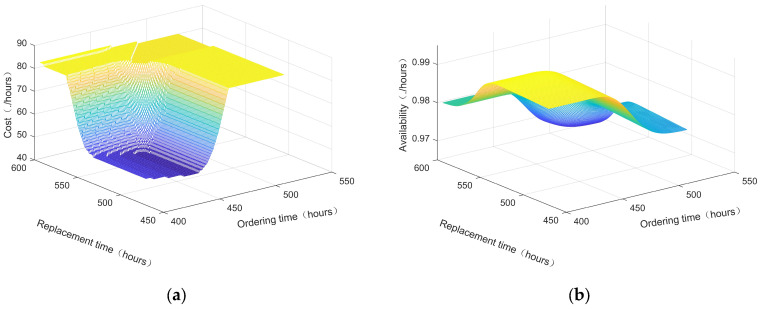
Constraints: (**a**) Long-term average cost model; (**b**) Long-term average availability model.

According to the proposed method in Section 3, taking the 135th monitoring cycle at the current monitoring time point, i.e., the 405th hour, the constraint function graphs of long-term average cost and long-term average availability as a function of spare parts ordering and replacement time are plotted in Figure 5a and Figure 5b, respectively.

**Figure 6:**
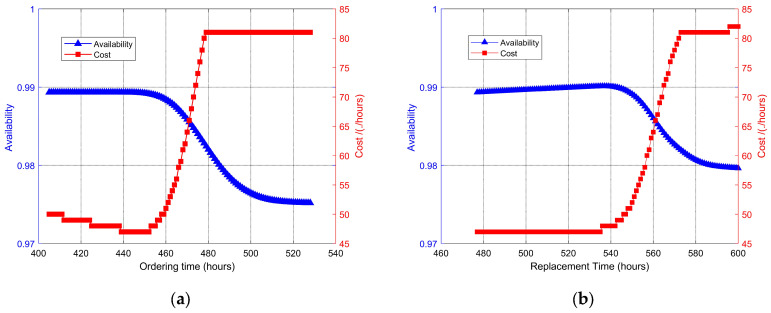
Maintenance decision-making: (**a**) ordering time; (**b**) replacement time.

To enhance the intuitiveness of the proposed maintenance decision-making results, Figure 6 presents these results. The blue line indicates the long-term average availability, while the red line represents the long-term average cost.

Figure 6 illustrates that the maintenance decision-making model identifies the optimal times for ordering spare parts and replacing components as the 439th hour and the 477th hour, respectively. This is attributed to the fact that the degradation of components tends to accelerate with increased operating time, leading to a gradual decline in their availability. The model developed in this article incorporates both cost and availability as key decision objectives, constructing a multi-objective decision function based on intelligent prognostics to determine the ideal times for spare parts ordering and component replacement.

### 5.3. Compared with Single Cost Decision-Making

**Figure 7:**
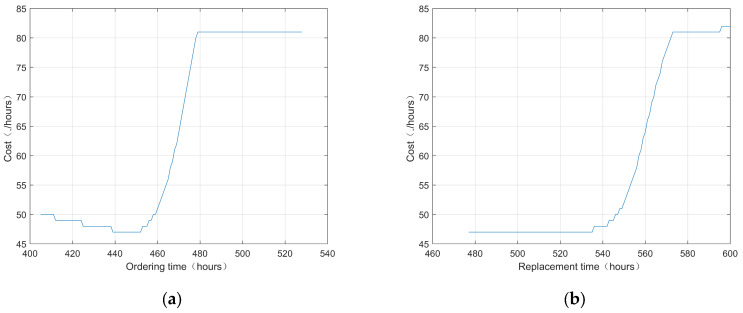
Single-cost decision: (**a**) ordering time; (**b**) replacement time.

To illustrate the importance of availability, only cost is chosen as the decision variable under identical conditions. Figure 7a,b present two-dimensional schematic diagrams of the decision outcomes from the single-cost decision-making model.

From Figure 7, it is evident that at the current monitoring time point of the 405th hour, if the objective is solely to minimize costs, the optimal times for ordering spare parts and replacing components are the 442nd and 480th hours, respectively. As the components continue to operate and accumulate running time, their degradation characteristics intensify, leading to a gradual decrease in availability as they near failure. Therefore, to ensure compliance with the required availability thresholds, it is essential to schedule spare parts ordering and replacement times in advance.

**Figure 8:**
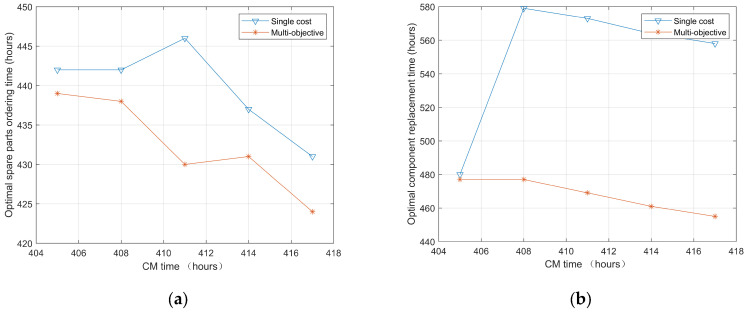
Optimal time: (**a**) spare parts ordering time; (**b**) component replacement time.

Similarly, in order to evaluate the generalization ability of the proposed model, a comparative analysis was conducted at different CM time points (e.g., 405, 408, 411, 414, and 417), and the results are shown in Figure 8 and recorded in Table 2. Compared with the single-cost decision-making model that aims to minimize a single cost, the optimal spare parts ordering and replacement time obtained through the proposed multi-objective maintenance decision-making model in this article are both in advance, which can help operation and maintenance personnel arrange spare parts ordering and replacement activities in a timely manner, ensuring that components can efficiently and stably complete tasks.

## 6. Conclusions

This article presents a maintenance decision-making method that utilizes intelligent prognostics to address the practical needs of maintenance costs and the actual performance of components in a system that relies on a single inventory of spare parts during operation and maintenance processes. The primary focus of this study includes

1. Utilizing the predicted remaining useful life information to develop a maintenance decision-making method for spare parts ordering and component replacement that balances high availability with low cost.
2. Considering both cost and availability as key decision objectives, while treating spare parts ordering and component replacement time as decision variables. The method addresses the trade-off between these two objectives by establishing a decision boundary.
3. Aiming to minimize costs while still meeting availability requirements, the method determines the optimal time for ordering spare parts and replacing multiple degraded components.

Finally, the effectiveness of the proposed method was validated using an aircraft engine dataset. The decision-making results can assist operation and maintenance personnel in effectively planning spare parts and replacement activities, demonstrating a valuable application in engineering. In the future, we will further consider providing feedback on the decision-making results of intelligent prognostics.

## Figures and Tables

**Table 1:** C-MAPSS dataset.

Sub-Dataset	Training Engines	Testing Engines	Scenarios	Fault Modes
FD001	100	100	1	1
FD002	260	260	6	1
FD003	100	100	1	2
FD004	249	249	6	2

**Table 2:** Decision results of the two methods at different condition monitoring time points.

Model	Condition Monitoring Time	Optimal Spare Parts Ordering Time	Optimal Component Replacement Time	Maintenance Cost
Single-cost maintenance decision-making model	405	442	480	47
	408	442	579	47
	411	446	573	47
	414	437	564	47
	417	431	558	47
The proposed multi-objective maintenance decision-making model	405	439	477	47
	408	438	477	47
	411	430	469	47
	414	431	461	47
	417	424	455	47

## Data Availability

The data that support the findings of this study are available from the corresponding author upon reasonable request.

## Abbreviations

The following abbreviations are used in this manuscript:

RUL	Remaining useful life
PM	Predictive maintenance
PDF	Probability density function
CM	Condition monitoring
CDF	Cumulative distribution function
RF	Reliability function
